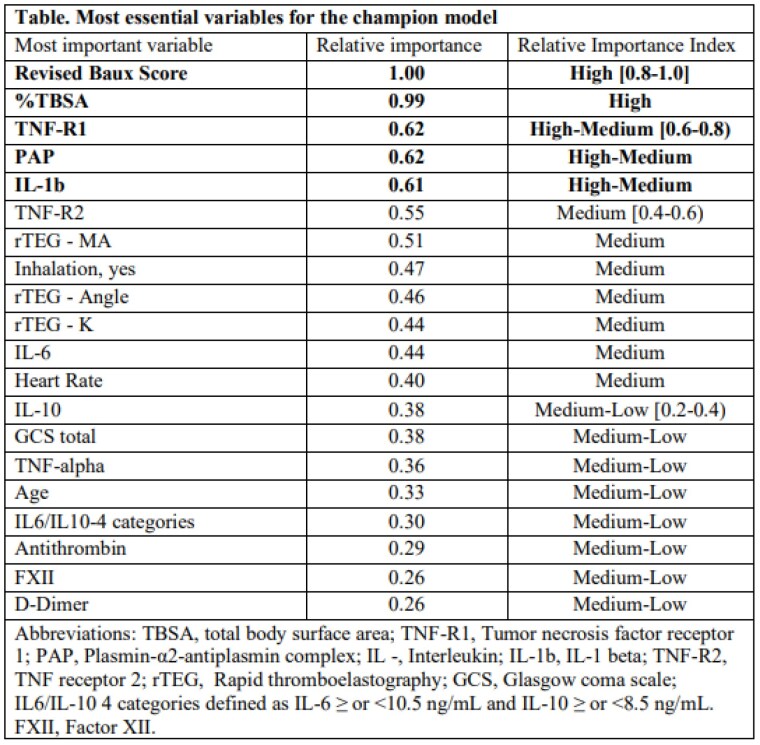# 53 Utilizing a Machine Learning Approach for the Prediction of In-Hospital Mortality After Thermal Burn

**DOI:** 10.1093/jbcr/iraf019.053

**Published:** 2025-04-01

**Authors:** Tuan Le, Amanda Soo Ping Chow, Anthony Pusateri, Melissa McLawhorn, Lauren Moffatt, Jeffrey Shupp

**Affiliations:** MedStar Health Research Institute; Burn Center at MedStar Washington Hospital Center; Naval Medical Research Unit San Antonio; MedStar Health Research Institute; MedStar Health Research Institute; MedStar Washington Hospital Center

## Abstract

**Introduction:**

Burn injury is a devastating form of trauma that can lead to long-term poor outcomes and death. Early and accurate mortality prediction is crucial for determining resuscitation status and determining appropriateness of care. This is especially important in situations of mass causalities where triage and resource availability are depleted quickly. This study focuses on the role of machine learning (ML) in predicting mortality, a system that has been increasingly used and proven effective in predicting clinical outcomes. The study’s aim was to identify ML models with the best diagnostic performance for predicting mortality in patients with burn injury.

**Methods:**

A retrospective observational study of 115 patients admitted to a regional burn center within 4 hours of thermal injury was conducted. Eighty-four features were selected, including patient demographic data, vital signs, injury characteristics- (%TBSA, inhalation injury, GCS, revised Baux score), CBC, vital signs, rapid thromboelastography, serum cytokine levels, coagulation markers, and clinical outcomes (e.g. mortality and hospital length of stay). Six ML models, including decision tree (DT), gradient boosting (GB), logistic regression (LR), neural network (NN), random forest (RF), and support vector machine (SVM) were identified to predict mortality. The ML models were compared by the area under the receiver operating characteristics curve (AUC) and Kolmogorov-Smirnov statistics (KS; Youden) for the best diagnostic performance for predicting mortality in burn patients, ensuring a comprehensive and rigorous approach to the analysis.

**Results:**

Of 115 patients studied, most were male (68.7%) with a median age of 40 years and TBSA of 11.8%, and 13 resulted in mortality (11.3%). The AUC values for the DT, GB, LR, NN, RF, and SVM models were 0.95, 0.99, 0.55, 0.99, 0.99, and 0.99, respectively. The KS values were 0.99, 0.99, 0.91, 0.99, 0.99, and 0.99, respectively. The SVM shows superior AUC and KS among these models, with sensitivity and specificity of 88% and 100%, respectively. The five essential predictors identified from the 20 most important variables for the champion model (Table) were revised Baux score, %TBSA, TNF-R1, PAP, and IL-1b.

**Conclusions:**

The SVM was the best-performing ML model (highest AUC and KS without overfitting), making it more reliable in predicting mortality. These findings, with the potential to significantly impact clinical practice, underline the importance of this research in burn injury management.

**Applicability of Research to Practice:**

Machine learning models, which have important applications in predicting patient outcomes, can provide real-time predictions of mortality and recovery, helping clinicians make more informed decisions in medical management.

**Funding for the Study:**

DoD Award Numbers W911NF-10-1-0459 and W911QY-15-C-0025